# Supplementary material for: Human Milk Oligosaccharide Profile Variation Throughout Postpartum in Healthy Women in a Brazilian Cohort
Source: Nutrients. 2020 Mar 17;12(3):790. doi: 10.3390/nu12030790 (PMC7146368; doi:10.3390/nu12030790)
Supplement: Supplementary file 1 [file nutrients-12-00790-s001.pdf]

Online Supplementary Material

Table S1. Time variation of HMOs concentrations according to Secretor status of women followed on Brazilian cohort. Rio de Janeiro, Brazil (n=101).

**HMOs (mmol/L)**	**Secretors women – median (IQR) Days postpartum (n)**			**Non-Secretors women – median (IQR) Days postpartum (n)**		
	**2-8 (n=46)**	**28-50 (n=68)**	**88-119 (n=42)**	**2-8 (n=6)**	**28-50 (n=7)**	**88-119 (n=4)**
			**Fucosylated or sialylated lactose**			
2'FL	5.42 (4.20-6.64)	5.18 (3.65-5.96)	4.38 (3.50-6.26)	0.01 (0.01-0.01)	0.03 (0.00-0.05)	0.03 (0.00-0.06)
3FL	0.28^{y} (0.19-0.46)	0.36^{z} (0.28-0.90)	2.24^{yz} (0.36-5.29)	0.13 (0.06-0.14)	0.16 (0.13-0.27)	1.02 (0.64-2.68)
DFLac	0.27^{y} (0.21-0.35)	0.39^{z} (0.31-0.52)	0.45^{yz} (0.39-0.60)	0.07 (0.01-0.13)	0.05 (0.03-0.07)	0.05 (0.01-0.10)
3'SL	0.32^{y} (0.25-0.48)	0.39^{z} (0.29-0.62)	0.55^{yz} (0.44-0.70)	0.20 (0.11-0.25)	0.33 (0.25-0.40)	0.35 (0.17-0.50)
6'SL	0.39^{xy} (0.22-0.55)	0.64^{xz} (0.49-0.93)	0.51^{yz} (0.39-0.74)	0.25 (0.23-0.55)	0.55 (0.43-0.89)	0.41 (0.31-0.59)
			**Non-fucosylated, non-sialylated HMOs**			
LNT	1.32 (1.02-1.92)	1.39 (1.03-1.96)	1.40 (1.09-1.72)	2.91 (2.47-3.33)	1.89 (1.53-2.49)	0.97 (0.81-1.07)
LNnT	0.58^{x} (0.30-1.15)	0.27^{xz} (0.17-0.41)	0.36^{z} (0.17-0.61)	0.62 (0.51-1.15)	0.30 (0.19-0.43)	0.26 (0.19-0.34)
LNH	0.06^{y} (0.03-0.11)	0.08^{z} (0.05-0.15)	0.06^{yz} (0.04-0.09)	0.11 (0.08-0.13)	0.08 (0.08-0.11)	0.07 (0.04-0.09)
			**Fucosylated, non-sialylated HMOs**			
LNFP I	2.85^{x} (2.27-3.49)	1.72^{xz} (1.06-2.42)	0.82^{z} (0.53-1.63)	0.10 (0.07-0.11)	0.12 (0.10-0.13)	0.13 (0.10-0.14)
LNFP II	0.75^{y} (0.59-1.02)	1.08^{z} (0.81-1.40)	1.55^{yz} (1.39-2.05)	2.17 (1.93-2.62)	2.52 (2.27-2.95)	3.37 (2.98-3.71)
LNFP III	0.08^{x} (0.07-0.12)	0.07^{xz} (0.05-0.10)	0.04^{z} (0.02-0.06)	0.08 (0.07-0.13)	0.08 (0.05-0.09)	0.05 (0.03-0.08)
DFLNT	1.54^{y} (1.00-1.85)	1.55^{z} (0.98-1.87)	1.27^{yz} (1.00-1.63)	0.30 (0.28-0.44)	0.39 (0.16-0.68)	0.92 (0.65-1.11)
FLNH	0.12^{y} (0.06-0.16)	0.15^{z} (0.08-0.23)	0.10^{yz} (0.07-0.14)	0.03 (0.02-0.08)	0.08 (0.04-0.13)	0.08 (0.07-0.10)
DFLNH	0.14 (0.06-0.22)	0.17 (0.10-0.26)	0.13 (0.05-0.21)	0.02 (0.01-0.03)	0.02 (0.01-0.03)	0.02 (0.01-0.09)
			**Non-fucosylated, sialylated HMOs**			
LSTb	0.08 (0.06-0.11)	0.10 (0.07-0.13)	0.07 (0.05-0.11)	0.18 (0.13-0.18)	0.16 (0.14-0.19)	0.11 (0.07-0.18)
LSTc	0.80^{x} (0.64-0.97)	0.27^{xz} (0.19-0.36)	0.11^{z} (0.07-0.16)	0.65 (0.43-0.71)	0.12 (0.10-0.35)	0.03 (0.02-0.04)
DSLNT	0.58^{x} (0.39-0.73)	0.30^{xz} (0.12-0.41)	0.18^{z} (0.03-0.30)	0.69 (0.43-0.86)	0.12 (0.07-0.39)	0.10 (0.02-0.27)
DSLNH	0.22^{xy} (0.12-0.29)	0.22^{xz} (0.15-0.32)	0.09^{z} (0.05-0.13)	0.16 (0.10-0.22)	0.28 (0.16-0.47)	0.09 (0.06-0.11)
			**Fucosylated, sialylated HMOs**			
FDSLNH	0.06^{y} (0.03-0.11)	0.16^{z} (0.08-0.28)	0.14^{yz} (0.09-0.23)	0.12 (0.09-0.16)	0.54 (0.38-0.63)	0.44 (0.21-0.77)
**Total**	16.88 (15.98-17.54)	15.58 (15.06-16.55)	16.93 (15.44-17.50)	9.68 (9.61-10.48)	8.97 (8.51-9.25)	9.29 (8.78-10.47)

IQR: interquartile range. ^{x} indicates that the HMO concentration at visit 1 is significantly different ($p < 0.05$) from the concentration at visit 2, ^{y} indicates that the HMO concentration at visit 1 is significantly different ($p < 0.05$) from the concentration at visit 3 and ^{z} indicates that the HMO concentration at visit 2 is significantly different ($p < 0.05$) from the concentration at visit 3 according to Friedman post-hoc test. . DFLac, difucosyllactose; DFLNH, difucosyllacto-N-hexaose; DFLNT, difucosyllacto-N-tetrose; DSLNH, disialyllacto-N-hexaose; DSLNT, disialyllacto-Ntetraose; FDSLNH, fucodisialyllacto-N-hexaose; FLNH, fucosyllacto-N-hexaose; HMO, human milk

oligosaccharide; LNFP, lacto-N-fucopentaose; LNH, lacto-N-hexaose; LNnT, lacto-N-neotetraose; LNT, lacto-N-tetrose; LSTb, sialyl-lacto-N-tetraose b; LSTc, sialyl-lacto-N-tetraose c; 2'FL, 2'-fucosyllactose; 3FL, 3-fucosyllactose; 3'SL, 3'-sialyllactose; 6'SL, 6'-sialyllactose.
